# Targeting neurosteroidogenesis as therapy for PTSD

**DOI:** 10.3389/fphar.2013.00166

**Published:** 2014-01-06

**Authors:** Graziano Pinna

**Affiliations:** Psychiatric Institute, Department of Psychiatry, University of Illinois at ChicagoChicago, IL, USA

**Keywords:** neurosteroidogenesis, PTSD, PTSD treatment, GABAergic neurotransmission, anti-PTSD drug discovery, selective brain steroidogenic stimulants (SBSSs), allopregnanolone, ganaxolone

Posttraumatic stress disorder (PTSD) is a severe condition resulting from exposure to traumatic events, such as combat situations, sexual assault, serious injury or the threat of death. Symptoms include disturbing recurring flashbacks, avoidance or numbing of memories of the event, and hyperarousal, which continue for more than a month after the traumatic event. Reduced cortical GABA (Kugaya et al., 2003) and cebrospinal fluid (CSF) allopregnanolone levels (Rasmusson et al., 2006) that positively and allosterically modulate GABA action at GABA_A_ receptors (Belelli and Lambert, 2005) suggest that in PTSD patients, a perturbation of GABAergic neurotransmission plays a role in the pathogenesis of this disorder. Thus restoring downregulated brain allopregnanolone levels may be beneficial in treating PTSD.

There is a general consensus that maladaptive fear responses (i.e., impaired fear extinction) are a core feature of stress-induced PTSD (Myers and Davis, 2007; Maren, 2008). Exaggerated fear responses and impaired extinction learning, or the inability to extinguish fear memories, are often treated with exposure-based therapy (EBT), which involves the exposure of the patient to the feared context without any danger (Joseph and Gray, 2008). This closely approximates the procedure used to simulate and study fear responses and fear extinction learning in PTSD mouse models (Marks, 1979). While psychological therapy has been highly effective both in treating PTSD and in preventing the progression of the event sequelae that leads to consolidation of fear memories, one challenge of PTSD therapy is the spontaneous recovery of fear that often reemerges following successful EBT.

For this reason, pharmacological treatment may be advantageous alone or in combination with EBT. Selective serotonin reuptake inhibitors (SSRIs) are currently the drugs of choice in treating PTSD. They are effective in facilitating and restoring the neurobiological changes altered in PTSD patients, and they are devoid of the unwanted side effects that plague the use of benzodiazepines, more importantly, SSRIs are potent therapeutics where benzodiazepines fail to be beneficial. Following the observation that low non-serotonergic doses of fluoxetine and congeners increase allopregnanolone levels as their primary mechanism of action, we suggested that SSRIs acting as selective brain steroidogenic stimulants (SBSSs) can improve dysfunctional emotional behavior and may be of advantage in PTSD treatment. In addition to its use in PTSD, this novel steroidogenic mechanism of action of SSRIs given at low doses offers enormous therapeutic potentials for the treatment of other psychiatric disorders, including anxiety spectrum disorders, premenstrual dysphoria, and probably depression, as these disorders may be caused by a downregulation of neurosteroid biosynthesis (Uzunov et al., 1996; Westenberg, 1996; Guidotti and Costa, 1998; Romeo et al., 1998; Uzunova et al., 1998; Steiner and Pearlstein, 2000; Berton and Nestler, 2006; Pinna et al., 2006a, 2009; Pinna, 2010; Ipser and Stein, 2012; Pinna and Rasmusson, 2012; Lovick, 2013).

In vitro studies show that SSRIs may activate 3α-hydroxysteroid dehydrogenase, thereby facilitating the reduction of 5α-dihydroprogesterone into allopregnanolone (Griffin and Mellon, 1999). Nonetheless, the precise neuronal mechanisms involved in the neurosteroidogenic action of SSRIs remain unclear. Drug design welcomed allopregnanolone biosynthesis as a target for novel rapidly acting anxiolytics devoid of sedation, tolerance, and withdrawal liabilities (Rupprecht et al., 2009, 2010; Schüle et al., 2011), and, in addition to low doses of SSRIs, selective ligands for the (18 kDa) translocase protein (TSPO), which increase allopregnanolone levels, may be beneficial in anxiety and PTSD (Rupprecht et al., 2009).

## A PTSD mouse model

In our laboratory, we have used the socially isolated (SI) mouse as a model characterized by a downregulation of allopregnanolone biosynthesis associated with endophenotypic features of PTSD. The relevance of the SI mice as a model of PTSD lays in reproducing behavioral and neurochemical alterations that are found in PTSD patients (Pibiri et al., 2008). Thus, SI mice express decreased corticolimbic allopregnanolone levels in emotion-relevant brain areas (frontal cortex, hippocampus, basolateral amygdala) (Pibiri et al., 2008; Pinna et al., 2008). The impulsivity and violence of combat veterans (Forbes et al., 2008), is matched in SI mice by high levels of aggression (Pinna et al., 2003). In PTSD patients, enhanced contextual fear and impaired fear extinction learning was shown during re-exposure to events that symbolize the triggering traumatic event; however, cued fear was not changed (Ameli et al., 2001; Rauch et al., 2006). SI mice, analogously, display exaggerated contextual fear and impaired fear extinction and unchanged cued fear responses (Pibiri et al., 2008; Pinna et al., 2008). Interestingly, PTSD patients fail to respond to the pharmacological action of benzodiazepine and show decreased frontocortical benzodiazepine site binding (Bremner et al., 2000). Of note, SI mice show a lack of sedative/anxiolytic activity to diazepam and zolpidem (Pinna et al., 2006b; Nin et al., 2011). In contrast, allopregnanolone or S-norfluoxetine at low, non-SSRI active doses reduced anxiety in SI mice, an effect that was mimicked by allopregnanolone's analog ganaxolone (Pinna and Rasmusson, submitted). Interestingly, anxiolytic doses of S-norfluoxetine also normalized the immobility time of SI mice as determined by the forced swim test (Nin et al., 2011).

Social isolation causes changes in the frontocortical and hippocampus expression of GABA_A_ receptor subunits. The cortical expression of α1, α2, and γ2 subunit mRNA was decreased by ≈50%, and α4 and α5 was increased by 130% in SI mice. The expression α1 subunit mRNA in layer I was decreased by 50% and unchanged in layer V of SI mice (Pinna et al., 2006b). Likewise, GABA_A_ receptor subunit expression of α1 was decreased and that of α5 was increased in the hippocampus. A downregulation of α1 (−40%) and an increase in the expression of α5 subunit proteins (+100%) was also determined in SI mice. Because γ2 subunits are a necessary prerequisite for the formation of benzodiazepine-sensitive GABA_A_ receptors, our study suggests that the decrease in γ2 expression and the lack of benzodiazepine's anxiolytic action observed in SI mice may be a result of stress-induced formation of benzodiazepine-insensitive GABA_A_ receptors strategically integrated in circuitry that regulate anxiety. Interestingly, we observed a decreased benzodiazepine binding to hippocampal synaptic membranes (Pinna et al., 2006b).

Unlike benzodiazepines, which have a selective pharmacological profile and fail to activate GABA_A_ receptors containing α4 and α6 subunits (Brown et al., 2002), allopregnanolone modulation of GABA_A_ receptors exhibits a broad pharmacological profile. Although allopregnanolone acts preferentially on δ subunit-containing GABA_A_ receptors, which confers neurosteroid sensitivity, it also exerts effects on other GABA_A_ receptor subtypes at higher concentrations (Mihalek et al., 1999; Stell et al., 2003). Thus, increasing corticolimbic allopregnanolone levels with allopregnanolone injections or stimulating allopregnanolone biosynthesis with S-norfluoxetine, or directly activation of GABA_A_ receptors with ganaxolone likely improved anxiety because allopregnanolone/ganaxolone acts on a larger spectrum of GABA_A_ receptor subunits. Thus, allopregnanolone or analogs are more advantageous than benzodiazepines because they improve anxiety, fear, and aggressiveness when benzodiazepines are inactive. In addition, unlike benzodiazepines, allopregnanolone, ganaxolone, or SBSS ligands may improve emotional behavior at non-sedative concentrations (Pinna et al., 2003, 2006b; Nelson and Pinna, 2011; Nin et al., 2011; Pinna and Rasmusson, submitted). These observations suggest that drugs designed to selectively increase neurosteroidogenesis may alleviate PTSD by facilitating GABA_A_ receptor neurotransmission.

## Pharmacological targets to stimulate neurosteroidogenesis

**Figure 1:**
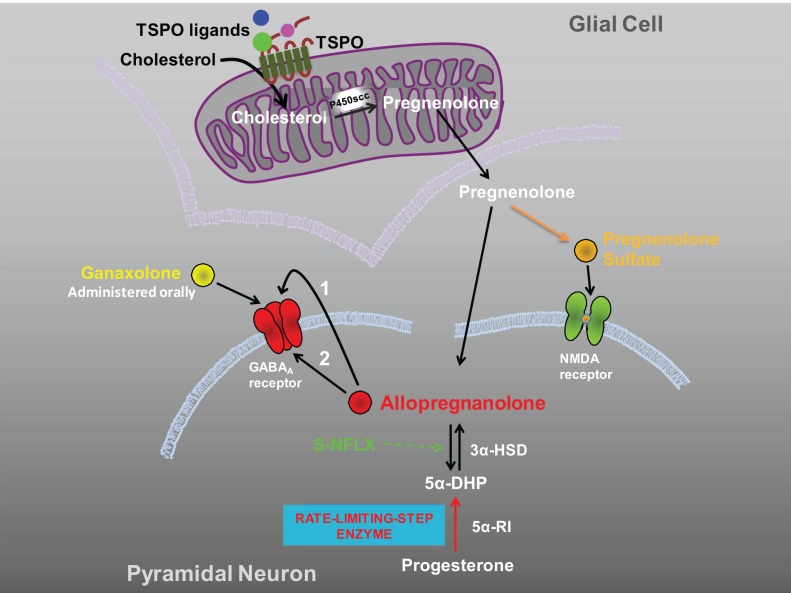
**Therapeutic strategies to increase neurosteroidogenesis and improve PTSD by enhancing GABAergic neurotransmission.** Depicted are three strategies to improve PTSD symptoms by increasing corticolimbic allopregnanolone levels or by direct activation of GABA_A_ receptors. (A) TSPO ligands induce an *upstream* regulation of neurosteroidogenesis by gating the entry of cholesterol into the inner mitochondrial membranes of glial cells, and its conversion into pregnenolone. Pregnenolone can then be taken up by pyramidal neurons (Costa and Guidotti, 1991) where a cascade of enzymatic processes takes place in the cytosol resulting in the production of allopregnanolone. Interestingly, pregnenolone can be further sulfated to pregnenolone sulfate, which has been described as both a positive NMDA receptor modulator (Kussius et al., 2009) and negative GABA_A_ receptor modulator (Mtchedlishvili and Kapur, 2003). (B) S-NFLX induces a *downstream* activation of neurosteroidogenesis likely by stimulating allopregnanolone content at the level of 3α-HSD (Griffin and Mellon, 1999). Neurosteroidogenesis is not globally expressed in the brain but relies on rate-limiting step enzymes, which guard allopregnanolone availability and thereby normalize its physiological levels in the required corticolimbic areas (e.g., after activation of TSPO or after S-NFLX). Allopregnanolone, synthesized in glutamatergic cortical or hippocampal pyramidal neurons, may improve PTSD symptoms after being secreted by an autocrine fashion and act locally by binding post-synaptic or extra-synaptic GABA_A_ receptors located on the same neuron in which it was produced (arrow 1) (Agis-Balboa et al., 2006, 2007). Allopregnanolone may also diffuse into synaptosome membranes of the cell bodies or dendritic arborization to attain intracellular access to specific neurosteroid binding sites of GABA_A_ receptors (arrow 2) (Akk et al., 2005). (C) Allopregnanolone's analogs (e.g., ganaxolone) directly activate GABA_A_ receptors and are beneficial in pathological conditions in which allopregnanolone biosynthesis is severely impaired. TSPO, translocate protein (18 kDa); 5α-DHP, 5α-dihydroprogesterone; 5α-RI, 5α-reductase type I; 3α-HSD, 3α-hydroxysteroid dehydrogenase; S-NFLX, S-norfluoxetine.

A seminal observation by Uzunova et al. (1998) suggested that SSRIs, including fluoxetine and fluvoxamine might be beneficial in the treatment of major unipolar depression by increasing the brain levels of allopregnanolone. This SSRI-induced neurosteroidogenic effect correlated with improved depressive symptomatology and was confirmed by several other reports in the field (Romeo et al., 1998, reviewed in Pinna et al., 2006a; Schüle et al., 2011). Previous studies reported that SSRIs induce allopregnanolone biosynthesis in rodent brain slices following incubation with the allopregnanolone's precursor 5α-dihydroprogesterone (Uzunov et al., 1996). These observations were confirmed in experiments in which fluoxetine's ability to induce neurosteroidogenesis in several corticolimbic structures was challenged using mouse models of psychiatric disorders such as the SI mouse (Pinna et al., 2003, 2004). Interestingly, fluoxetine's action as a steroidogenic stimulant appeared to be the primary mechanism of SSRIs: the drug concentrations, which increased brain allopregnanolone levels were less than, and dissociated from, those effective as a *selective serotonin reuptake inhibitor*, which justified a new name to better define the “SSRI” mechanism of action: *selective brain steroidogenic stimulants* or *SBSS* (Pinna et al., 2006a, 2009). The discovery of this novel mechanism of action of SSRIs has stimulated drug design to focus on the development of new, more effective therapies for anxiety disorders by targeting neurosteroidogenesis. Novel neuronal biomarkers, for the pharmacological target of neurosteroidogenesis as the next generation of anxiolytic drugs, have been discovered (Rupprecht et al., 2009). These include the TSPO (Costa et al., 1994; Papadopoulos et al., 2006), which represents the starting point and an important rate-limiting step in neurosteroidogenesis. TSPO regulates neurosteroidogenesis in the brain by gating the entry of cholesterol into the inner mitochondrial membranes of glial cells, and its conversion into pregnenolone by P450scc Figure 1 (Costa and Guidotti, 1991; Costa et al., 1994; Papadopoulos et al., 2006; Rupprecht et al., 2010). Pregnenolone can then be taken up by pyramidal neurons (Costa and Guidotti, 1991) where a cascade of enzymatic processes takes place in the cytosol resulting in the production of neurosteroids, including pregnenolone sulfate and allopregnanolone Figure 1. New molecules that bind with high affinity to TSPO have been recently investigated; these drugs are able to exert important anxiolytic effects but are devoid of the unwanted side effects associated with benzodiazepines, including over-sedation, tolerance, and withdrawal symptoms (Rupprecht et al., 2009, 2010). In mouse models, TSPO agents have been shown to potently increase allopregnanolone levels in the hippocampus and cortex, as well as to induce anxiolytic effects (Kita et al., 2004). XBD173 and etifoxine have proven to be highly efficacious anxiolytic and antidepressant drugs in a number of behavioral tests (Rupprecht et al., 2010; Schüle et al., 2011). The anxiolytic effects of these agents were related to their ability to increase neurosteroid biosynthesis *upstream* of allopregnanolone synthesis within the neurosteroidogenic cascade Figure 1, as confirmed by studies in which key enzyme blockers for neurosteroid biosynthesis, including finansteride and trilostane (Schüle et al., 2011), were used. TSPO ligands (AC-5216/XBD173 and YL-IPA08) also improve PTSD-like behavior in rodents in studies of situational reminders and contextual fear responses (Qiu et al., 2013). In summary, these studies demonstrated the neuropharmacological effects of several TSPO agents, suggesting that TSPO may represent a therapeutic target for drug discovery. Thus, these drugs, which fulfill the requirements as SBSS molecules, may be a new class of drugs for the future treatment of PTSD and other anxiety disorders. Consistently, TSPO ligands have recently showed promising therapeutic effects in clinical studies (Rupprecht et al., 2010; Schüle et al., 2011).

The advantage of having a drug that “indirectly” activates GABA_A_ receptors by increasing allopregnanolone levels Figure 1 within the brain is that allopregnanolone will not be globally increased. Physiological concentrations of allopregnanolone are unevenly expressed in the brain (Pinna et al., 2000; Pibiri et al., 2008), and regulated by rate-limiting step enzymes such as 5α-reductase type I. Pharmacological treatments also induce a cell specific upregulation of brain allopregnanolone, which is increased in frontal cortex (pyramidal neurons, 5α-reductase is not expressed in interneurons), hippocampus (CA1-3 pyramidal neurons and dentate gyrus granular cells), and basolateral amygdala (pyramidal-like neurons) after fluoxetine but not in striatum (where allopregnanolone is produced in GABAergic long-projecting neurons, spiny neurons) (Agis-Balboa et al., 2006, 2007). Hence, while allopregnanolone is downregulated during social isolation, fluoxetine elevates its levels in glutamatergic neurons but not in GABAergic neurons (Nelson and Pinna, 2011). If allopregnanolone is administered directly, it would be expressed all over the brain and reach high levels in brain regions where its levels are physiologically lower.

Ideally, the SBSS drugs of the future that selectively induce anxiolytic and anti-PTSD effects, will be those molecules, prototypic of fluoxetine, devoid of serotonergic effects but capable of activating a neurosteroidogenesis cascade *downstream*, possibly stimulating allopregnanolone content at the level of 5α-reductase or 3α-hydroxysteroid dehydrogenase. Understanding whether FLX's action on neurosteroidogenesis is mediated by upregulating expression or function of 5α-reductase is of pivotal importance because this enzyme is downregulated in corticolimbic areas of SI mice and in post-mortem frontal cortex (BA9) of depressed patients (Agis-Balboa et al., submitted).

As an alternative, in patients who cannot adequately synthesize allopregnanolone and in whom administration of an SBSS is ineffective because neurosteroidogenesis is greatly impaired, the administration of an allopregnanolone analog (Gulinello et al., 2003; Kaminski et al., 2004), such as ganaxolone that directly activates GABA_A_ receptors Figure 1 may offer a safe therapeutic alternative. A multisite Phase II trial of the efficacy and safety of ganaxolone in PTSD is currently under process.

## Conclusion

Targeting allopregnanolone biosynthesis with selective neurosteroidogenic agents offers several therapeutic advantages: (1) allopregnanolone is not globally expressed in the brain like in the case of administering allopregnanolone itself, in fact, using a neurosteroidogenic molecule relies on the stimulation of rate-limiting step enzymes Figure 1, which guard allopregnanolone levels and thereby normalize its physiological levels in the required brain areas; and (2) stimulating allopregnanolone biosynthesis downstream of pregnenolone in the neurosteroidogenic cascade circumvents the production of several neurosteroids, which by activating various neurotransmitter systems may be associated with unwanted side effects.
